# Separation of saturated fatty acids from docosahexaenoic acid‐rich algal oil by enzymatic ethanolysis in tandem with molecular distillation

**DOI:** 10.1002/fsn3.1462

**Published:** 2020-04-17

**Authors:** Jianlin He, Bihong Hong, Rong Lu, Ruoqi Zhang, Hua Fang, Wenwen Huang, Kaikai Bai, Jipeng Sun

**Affiliations:** ^1^ Third Institute of Oceanography Ministry of Natural Resources Xiamen China; ^2^ Technical Innovation Center for Utilization of Marine Biological Resources Ministry of Natural Resources Xiamen China; ^3^ Department of Laboratory Medicine The First Affiliated Hospital of Xiamen University Xiamen China; ^4^ School of Pharmacy Chengdu University of Traditional Chinese Medicine Chengdu China; ^5^ Zhejiang Marine Development Research Institute Zhoushan China

**Keywords:** DHA‐rich algal oil, ethanolysis, Lipozyme^®^ TL IM, molecular distillation

## Abstract

Algal oil, rich in docosahexaenoic acid (DHA) and an environmentally sustainable source of ω‐3 fatty acids, is receiving increasing attention. In the present study, a novel approach combining ethanolysis with a 1,3‐specific immobilized lipase (Lipozyme^®^ TL IM) and molecular distillation was investigated to increase the DHA content of algal oil. Algal oil with a 45.94% DHA content was mixed with ethanol, pumped into a column filled with Lipozyme^®^ TL IM, and then circulated for 4 hr at room temperature. The ethanol was then recycled by vacuum distillation. At an evaporator temperature of 150°C, the residue was separated by molecular distillation into a heavy component enriched with DHA glycerides (in the form of triglyceride (TG), diglyceride (DG), and monoglyceride (MG)) and a light component enriched with palmitic acid (PA) and DHA ethyl ester (EE). As a result, 76.55% of the DHA from the algal oil was present in the heavy component, whose DHA content was 70.27%. DHA‐MG was collected in the heavy component mostly in the form of 1‐MG. Lipozyme^®^ TL IM appeared to specifically target PA rather than DHA at the sn‐1(3) position. The Lipozyme^®^ TL IM allowed 90.03% of the initial DHA yield to be retained after seven reaction cycles. Therefore, an eco‐friendly and simple method for increasing the DHA content in algal oil has been developed.

## Graphical abstract

Ethanolysis and molecular distillation were tandem to separate saturated oil from algal oil to increase the DHA content from 45.9% to 70.3%. We found that DHA monoglyceride and ethyl ester can be separated by molecular distillation. Lipozyme^®^ TL IM might provide an ethanolysis specific to PA rather than DHA at the sn‐1(3) positions. The Lipozyme^®^ TL IM allowed 90.03% of the initial DHA yield to be retained after seven reaction cycles.

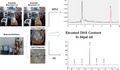

## 1 INTRODUCTION

Docosahexaenoic acid (DHA) is an essential fatty acid with many important physiological regulatory functions. DHA plays a vital role in nerve development in infants and young children, and in maintaining brain functions in adults (Lauritzen et al., 2016). Its other beneficial effects, such as reducing blood fat, blood pressure, and cholesterol, preventing neurological disease, and anti‐inflammatory activity (He, Huang, Zheng, Vigorito, & Chang, 2018), have also been well established.

DHA enrichment is of great interest for the producers of functional foods, healthcare products, and drugs. Fish oil commonly contains 12% DHA and 18% eicosapentaenoic acid (EPA). Currently, the ethyl ester (EE) form is widely used to enrich ω‐3 fatty acids in fish oil. The EE form is converted from the natural triglyceride (TG) form to obtain higher levels of EPA and DHA. However, DHA‐EE might not be fully absorbed owing to its low bioavailability. The absorption rate of DHA‐TG was found to be about 57% in humans, while that of DHA‐EE was only about 21% (Lawson & Hughes, 1988). DHA‐EE also produces ethanol as a metabolite, so is not suitable for children or adults with an alcohol allergy. DHA‐EE is reportedly less stable and produces more harmful oxidative byproducts than DHA‐TG (Ritter, Budge, Jovica, & Reid, 2015; Yoshii et al., 2002). In products derived from fish oil, it is difficult to avoid contamination with heavy metals, such as methylmercury (Silbernagel et al., 2011), and organic pollutants, such as polychlorinated biphenyls (Fernandes, Rose, White, Mortimer, & Gem, 2006). Furthermore, fish oil is not suitable for vegetarians. Therefore, DHA‐rich algal oil has advantages over mainstream fish oil products on the market in terms of absorption rate, stability, and safety.

Through heterotrophic culture, n‐3 biotechnological processes for DHA have been well established and been developed into industrial‐scale production. Commonly, algal oil obtained from *Schizochytrium* sp. has a DHA content of about 45% and occupies an increasing market share. For nutritional and pharmacological purposes, it is vital to further increase the DHA concentration. Urea complexation (Hayes, Bengtsson, Van Alstine, & Setterwall, 1998), low‐temperature crystallization (Brown, 1941), solvent extraction, and column chromatography, which require huge amounts of solvent, are widely used to concentrate or purify different forms of fatty acid.

In recent decades, lipase‐catalyzed reactions have been used to concentrate DHA and other n‐3 polyunsaturated fatty acids (PUFA), particularly fish oil. For example, enzymatic ethanolysis has been used to convert n‐3 PUFA to the corresponding EEs from low‐grade fish oil feedstocks (Yan et al., 2018). Oil extracted from Pacific oysters has been used for ethanolysis reactions catalyzed by Novozym^®^ −435, Lipozyme^®^ TL IM, and Lipozyme^®^ RMIM to produce 2‐MG rich in n‐3 PUFAs (Lee, Haq, Saravana, Cho, & Chun, 2017). A typical two‐step synthesis of DHA‐rich symmetrically structured TG was based on 2‐MG produced by the enzymatic ethanolysis of bonito oil (Irimescu, Furihata, Hata, Iwasaki, & Yamane, 2001), or cod liver and tuna oils (Munio, Robles, Esteban, Gonzalez, & Molina, 2009).

The present study will use enzymatic ethanolysis techniques to avoid the large‐scale use of toxic solvents. This environmentally friendly approach aims to combine enzymatic ethanolysis with molecular distillation to increase the DHA content of algal oil.

## 2 MATERIALS AND METHODS

### 2.1 Materials

DHA, DHA‐EE, DHA monoglyceride (DHA‐MG), and PA‐EE were purchased from Nu‐chek Prep Inc. (Elysian), HPLC‐grade acetonitrile, *n*‐hexane, and methanol from Merck and standards of 37 fatty acid methyl esters (FAMEs) from Sigma‐Aldrich. Algal oil from *Schizochytrium* sp. was provided by Xiamen Huisheng Group Co.. Lipozyme^®^ TL IM (a silica granulated *Thermomyces lanuginosus* lipase preparation, 250 U/g) was purchased from Novozymes A/S.

### 2.2 Enzymatic ethanolysis of DHA algal oil

The DHA‐rich algal oil was mixed well with ethanol then pumped into a column filled with Lipozyme^®^ TL IM and circulated using a peristaltic pump. The optimum conditions for algal oil ethanolysis were an algal oil/ethanol mass ratio of 1:2 at room temperature for 4 hr with 13% Lipozyme^®^ TL IM (w/w, relative to total reactants) as the catalyst, as determined by a previous study (He, Bai, Hong, Chen, & Yi, 2016). The product was then distilled under reduced pressure to recycle the ethanol. After each round of the ethanolysis reaction, the immobilized lipase was washed by pumping three column volumes of ethanol through the column. After removing the solvent, the recycled lipase was stored at 4°C before reuse in a new reaction.

### 2.3 Molecular distillation

KDL‐2 short path distillation equipment (UIC Corporation, Alzenau, Germany) was used in the present study. The algal oil was placed in the feed vessel, which was surrounded by a heating jacket. A temperature of 40°C was used to ensure that the oil flowed well. The vacuum pump was switched on after liquid nitrogen had been added to the cold trap. The evaporation pressure fell to below 1 × 10^−3^ mPa after the diffusion pump was turned on. The wiper basket was switched on at a low speed when the evaporator temperature rose. When the desired temperature had been reached, the speed of the wiper basket was set to 350 rpm. The feed vessel was then cautiously opened, and the oil allowed to flow dropwise into the evaporation system through the roller wiper system at a feed rate of approximately 80 ml/h. The light components were quickly vaporized and cooled on the condenser surface, and the distillate was collected. Almost nothing was found in the cold trap cooled with liquid nitrogen. Any heavy components that could not be vaporized were collected in a collecting bulb. After collection, all samples were kept below 4°C in the refrigerator.

### 2.4 Gas chromatography (GC) analysis

The FAMEs were prepared based on the AOCS method Ce‐1b 89 (2007). A 37‐component FAME mix from Sigma‐Aldrich was used as the FAME external standard, which was run in parallel with the samples.

The FAMEs were analyzed using an Agilent 7890B GC system with a flame ionization detector (Agilent Technologies) and a fused‐silica capillary column (Sp‐2560, 100 m × 0.25 mm × 0.2 μm, Supelco Inc.). The injection port and detector temperatures were both set at 250°C. The column temperature was initially ramped to 210°C at a rate of 3°C/min and held for 1 min, then ramped to 219°C at a rate of 0.5°C/min and held for 1 min, and finally ramped to 240°C at a rate of 10°C/min and held for 13.5 min. The final temperature was 250°C, which was held for 1 min. The GC peaks were identified by comparing the retention times with those of the corresponding standards, with the relative contents then calculated, and expressed in mol%.

The PA‐EE was analyzed under the same GC conditions. The concentrations of PA‐EE were calculated from the peak areas from GC using the corresponding linear regression equation in an external standard calibration method.

### 2.5 High‐performance liquid chromatography (HPLC) analysis

The analytes were separated on a Waters 2695 Alliance HPLC system with a 2998 PDA detector (Waters) using an Agilent C18 column (250 × 4.6 mm, 5 μm, Agilent Technologies). The mobile phase was composed of acetonitrile (A) and water containing 0.1% formic acid (B), (A/B = 90:10 or 70:30, v/v) at a flow rate of 1 ml/min with an injection volume of 5 μl. The detection wavelength was set at 205 nm. The concentrations of DHA‐EE and DHA‐MAG were calculated from the peak areas using the corresponding linear regression equation in an external standard calibration method.

### 2.6 Analysis of fatty acid composition at sn‐2 position

The fatty acid composition at the sn‐2 position of the algal oil was determined by pancreatic lipase‐catalyzed hydrolysis (Luddy, Barford, Herb, Magidman, & Riemenschneider, 1964; Pina‐Rodriguez & Akoh, 2009). Briefly, 1 M Tris‐HCl buffer (2 ml), 0.05% sodium cholate solution (0.5 ml), 2.2% calcium chloride solution (0.2 ml), and pancreatic lipase (40 mg) were mixed with algal oil (100 mg) then incubated at 40°C for 3 min. The mixture was then vortexed for 2 min, and 6 M HCl (1 ml) was added to stop the reaction. The hydrolyzed product was extracted twice with diethyl ether (2 ml), which was then removed by blowing with nitrogen gas. The concentrated extract was separated on a silicic acid 60 F_254_ TLC plate (Yantai Chemical Industry Research Institute) impregnated with a solution of boric acid in methanol (5%, w/v) to prevent isomerization, developed using hexane/diethyl ether/acetic acid (50:50:1, v/v/v), and visualized under UV light (254 nm). The band corresponding to 2‐MG was scraped off the TLC plate then converted to the corresponding FAME. The composition was then analyzed by GC.

### 2.7 Analysis of glyceride composition in heavy component

To analyze the glyceride species in the heavy component, the sample was separated on a silicic acid 60 F_254_ TLC plate, developed using hexane/diethyl ether/acetic acid (80:20:1, v/v/v), and visualized under UV light (254 nm). The bands corresponding to MG, DG, and TG were scraped off the TLC plate, converted to the corresponding FAME, and then analyzed by GC. The total peak areas were calculated for each glyceride. The relative content of each glyceride in the heavy component was then estimated by comparison with the total peak areas.

### 2.8 Data analysis

Results were obtained from experiments conducted in at least triplicate. Data presented in tables and figures are average values with standard deviations.

## 3 RESULTS

### 3.1 Total and sn‐2 fatty acid compositions of algal oil

Table 1 shows that the predominant fatty acid of algal oil was DHA, accounting for 45.94% of the total fatty acids. The percentage of DHA located at the sn‐2 position of algal oil was 55.43%. The fatty acid composition of the sn‐1(3) position was calculated based on the total TG and sn‐2 position fatty acid compositions, according to the formula (3 × TG‐sn‐2 MG)/2 (Turon, Bachain, Caro, Pina, & Graille, 2002). Palmitic acid (PA) was also an abundant fatty acid in algal oil, accounting for 37.40% of the total fatty acids, mostly distributed at the sn‐1 or sn‐3 positions. Algal oil also contained a high level of n‐6 docosapentaenoic acid (DPA, 8.93%), a polyunsaturated fatty acid, which was also found to be preferentially located at the sn‐2 position.

**Table 1:** Total, sn‐2, and sn‐1(3) fatty acid compositions of DHA‐rich algal oil (*n* = 3)

Fatty acids	Total fatty acid (%)	Sn‐2 fatty acid (%)	Sn‐1(3) fatty acid (%) (Calculated)
14:0	0.60 ± 0.02	0.34 ± 0.02	0.73 ± 0.03
14:1 *n*−5	0.52 ± 0.01	n.d.	0.80 ± 0.02
16:0	37.40 ± 0.20	21.91 ± 0.06	45.14 ± 0.29
16:1 *n*−7	0.34 ± 0.01	n.d.	0.51 ± 0.02
18:0	1.43 ± 0.05	0.66 ± 0.01	1.81 ± 0.07
18:1 *n*−9	0.45 ± 0.02	0.31 ± 0.01	0.51 ± 0.03
20:5 *n*−3	0.57 ± 0.01	0.20 ± 0.02	0.75 ± 0.01
22:5 *n*−6	8.93 ± 0.06	14.52 ± 0.11	6.14 ± 0.09
22:5 *n*−3	0.75 ± 0.02	0.54 ± 0.03	0.86 ± 0.02
22:6 *n*−3	45.94 ± 0.34	55.43 ± 0.81	41.19 ± 0.41

### 3.2 Reusability of Lipozyme^®^ TL IM in ethanolysis

**Figure 1:**
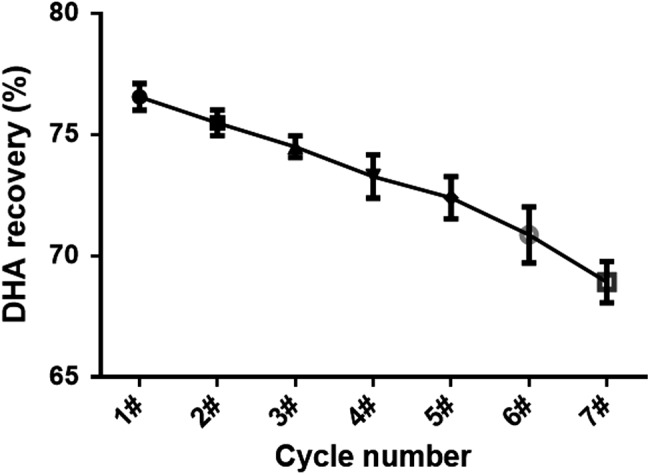
Reuse of Lipozyme^®^ TL IM in the ethanolysis of DHA‐rich algal oil. Experimental conditions: Algal oil/ethanol mass ratio of 1:2 at room temperature for 4 hr with 13% Lipozyme^®^ TL IM (w/w, relative to total reactants) (*n* = 3)

The reusability of the immobilized lipase was crucial for the feasibility of this bioprocess and the practical application of lipase. To evaluate the potential of the lipase for industrial application, the operational stability of Lipozyme^®^ TL IM was determined by measuring the DHA recovery in the heavy component obtained from successive ethanolysis reactions under the optimal conditions. DHA recovery was defined as the percentage of total DHA from the algal oil in the heavy component. After using the Lipozyme^®^ TL IM seven times, the DHA yield in the heavy component decreased from 76.55% to 68.92%, a retention of 90.03%, indicating that the lipase activity exhibited good stability (Figure 1). Therefore, Lipozyme^®^ TL IM showed good operational stability during enzymatic ethanolysis.

### 3.3 Molecular distillation

The PA, DPA, and DHA percentages in the light and heavy components under different molecular distillation conditions were determined by GC (Table 2). The evaporator temperature significantly affected the PA content in the light component and the DHA content in both the light and heavy components. The internal condenser temperature was set at 50°C and a pressure of ≤1 × 10^−3^ mPa. The yields, in terms of the percentage weight of the total distillate in each component, are also listed in Table 2. With increasing evaporator temperature, the yield of the heavy component decreased rapidly, while the yield of the light component increased. The recoveries of PA in the light component and DHA in the heavy component were also calculated. The DHA content reached up to 70% when the evaporator temperature was set at 150°C or higher. The DHA recovery in the heavy component was 76.55%, meaning that 76.55% of DHA from the algal oil was present in the heavy component.

**Table 2:** Fatty acid compositions of the light and heavy components at different evaporator temperatures (*n* = 3)

Evaporator (°C)	Light component					Heavy component				
	Yield (%)	Fatty acid composition (%)			PA recovery (%)	Yield (%)	Fatty acid composition (%)			DHA recovery (%)
		PA	DPA	DHA			PA	DPA	DHA	
100	23.7 ± 1.51	71.49 ± 0.69	0.59 ± 0.01	2.17 ± 0.20	77.58 ± 0.53	71.5 ± 0.31	6.85 ± 0.05	17.08 ± 0.65	66.94 ± 1.88	98.94 ± 0.74
120	36.2 ± 0.75	51.74 ± 0.27	4.80 ± 0.12	18.17 ± 1.16	85.12 ± 0.82	59.4 ± 0.79	5.51 ± 0.14	17.80 ± 0.32	69.15 ± 1.43	86.20 ± 0.88
150	43.5 ± 2.01	44.16 ± 1.04	6.93 ± 0.45	25.59 ± 0.48	87.27 ± 1.20	51.7 ± 0.98	5.42 ± 0.15	17.85 ± 0.46	70.27 ± 0.81	76.55 ± 1.02
180	54.8 ± 2.36	35.47 ± 0.89	9.18 ± 0.36	34.67 ± 0.78	88.36 ± 1.64	41.9 ± 1.54	6.11 ± 0.24	17.52 ± 0.23	69.95 ± 2.38	60.67 ± 1.49
200	60.2 ± 3.21	32.95 ± 0.76	10.17 ± 0.37	39.16 ± 2.34	90.12 ± 2.22	35.4 ± 1.02	6.14 ± 0.07	17.53 ± 0.20	70.07 ± 0.45	51.27 ± 2.18

### 3.4 HPLC determination of DHA‐EE and DHA‐MG in products of molecular distillation

**Figure 2:**
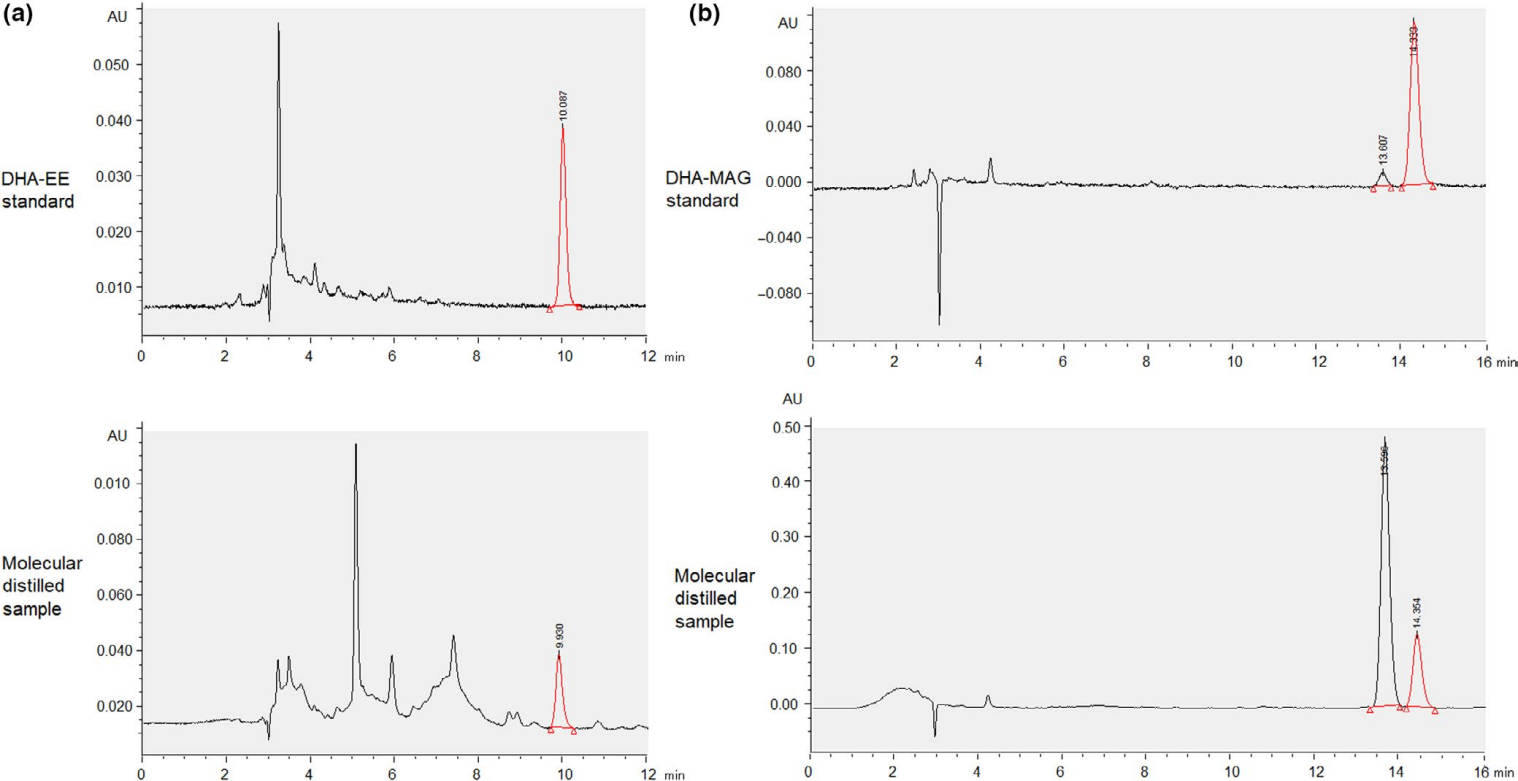
HPLC chromatograms of (a) DHA‐EE, where red the peak indicates DHA‐EE with a retention time of about 10.09 min, and (b) DHA‐MG, where the red peak indicates sn‐2 DHA‐MG with a retention time of about 14.33 min

Figure 2 shows that the retention time of DHA‐EE was about 10.09 min using a mobile phase of 90:10 (v/v) acetonitrile/0.1% formic acid with a flow rate of 1 ml/min. The DHA‐EE was well separated. The retention times of sn‐1/sn‐2 DHA‐MG were about 13.61/14.33 min, respectively, using a mobile phase of 70:30 (v/v) acetonitrile/0.1% formic acid with a flow rate of 1 ml/min. The HPLC method showed good linearity for DHA‐EE concentrations between 20 and 1,000 μg/mL and for DHA‐MG concentrations between 20.8 and 1,040 μg/mL (sn‐1 and sn‐2 DHA‐MG combined).

As part of the complex mixture of different forms of fatty acid in the molecular distillation products, the contents of DHA‐MG and DHA‐EE in the light and heavy components using different evaporator temperatures were determined by HPLC and the recovery was calculated (Table 3).

**Table 3:** Recovery of DHA‐MG and DHA‐EE in the light and heavy components of molecular distillation (*n* = 3)

Evaporator (°C)	Internal condenser (°C)	Pressure (mPa)	DHA‐MG recovery (%)		DHA‐EE recovery (%)	
			Light component	Heavy component	Light component	Heavy component
150	50	1 × 10^–3^	20.54 ± 0.14	79.58 ± 0.69	98.40 ± 0.60	1.60 ± 0.28
180	50	1 × 10^–3^	81.68 ± 1.81	18.32 ± 0.14	100.00 ± 0.00	0.00 ± 0.00
200	50	1 × 10^–3^	95.97 ± 0.44	4.03 ± 0.08	100.00 ± 0.00	0.00 ± 0.00
220	50	1 × 10^–3^	97.39 ± 0.45	2.61 ± 0.12	100.00 ± 0.00	0.00 ± 0.00

### 3.5 GC determination of PA‐EE in products of molecular distillation

**Figure 3:**
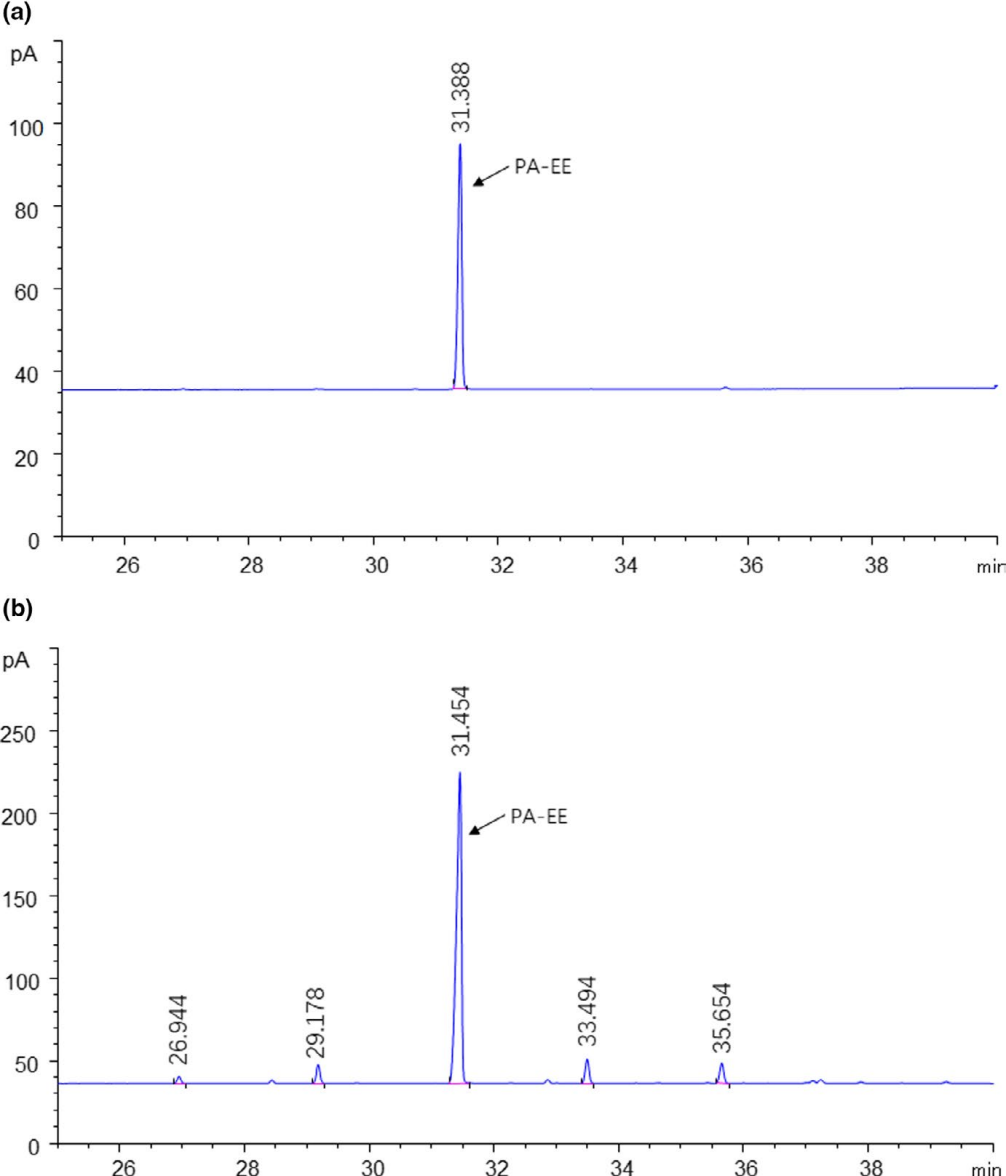
GC chromatograms of (a) PA‐EE standard and (b) PA‐EE in the molecular distilled samples

Figure 3 shows that the PA‐EE was well separated under the GC conditions, with a retention time of about 31.39 min. The GC method showed good linearity for PA‐EE concentrations between 100 and 1,000 μg/mL. The PA‐EE content in the light and heavy components and in the total reactants was determined by GC (Table 4).

**Table 4:** PA‐EE content in total reactants of ethanolysis, light component and heavy component of molecular distillation (*n* = 3)

PA‐EE content (%, w/w)		
Total reactant	Light component	Heavy component
24.05 ± 1.42	73.53 ± 3.20	2.55 ± 0.27

### 3.6 Glyceride composition in the heavy component

**Figure 4:**
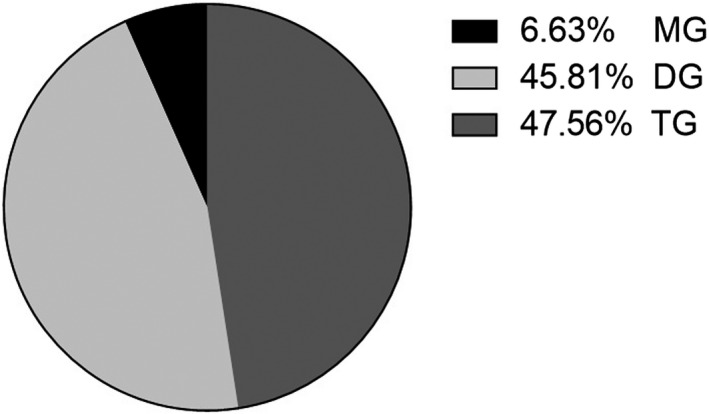
Composition of glyceride in the heavy component of molecular distillation

As shown in Figure 4, the glycerides in the heavy component consisted of 6.63% MG, 45.81% DG, and 47.56% TG.

## 4 DISCUSSION

As saturated fatty acids were found to be located mainly at the sn‐1 or sn‐3 position of the glycerol backbone of TG (Table 1), they were successfully removed by enzymatic ethanolysis with Lipozyme^®^ TL IM. The saturated fatty acids, with relatively low boiling points, were then evaporated by molecular distillation and concentrated in the light component. The dominant saturated fatty acid in the algal oil was PA, which was mostly present as PA‐EE in the light component (Table 4). The DHA was concentrated in the forms of MG, DG, and TG in the heavy component.

No solvents other than ethanol were used in this reaction. As both ethanol and Lipozyme^®^ TL IM can be recycled, our study has established an environmentally friendly method for increasing the DHA content of algal oil.

The preferential distribution of DHA at the sn‐2 positions in the TG form in algal oil should favor a large amount of sn‐2 DHA‐MG being present in the final product. Unexpectedly, more sn‐1(3) DHA‐MG than sn‐2 DHA‐MG was found in the distilled sample (Figure 2). Acyl migration from the sn‐2 position to the sn‐1(3) position has been reported to occur spontaneously, reaching a molar proportion of about 9:1 between 1‐MG and 2‐MG (Boswinkel, Derksen, Riet, & Cuperus, 1996; Sanchez, Tonetto, & Ferreira, 2016). A high temperature during molecular distillation also favors this migration (Ferreira & Tonetto, 2017; Poisson, Devos, Godet, Ergan, & Pencreac'h, 2009). At an evaporator temperature of 150°C, the sn‐1/sn‐2 DHA‐MG ratio was 3.92 in the heavy component, but 6.45 in the light component. This seemed reasonable considering that DHA‐MG in the light component experienced a high temperature for a longer time in the distillation plant.

Lipozyme^®^ TL IM is a 1,3‐specific lipase originating from *Thermomyces lanuginosus* and is immobilized on a noncompressible silica gel carrier (DiCosimo, McAuliffe, Poulose, & Bohlmann, 2013). Lipozyme^®^ TL IM is a highly effective catalyst for interesterification and can be used to cleave fatty acids at the sn‐1(3) position of the glycerol backbone. Lipozyme^®^ 435 can reportedly be recycled 5–10 times without an obvious loss in activity (Zhang et al., 2018). As Lipozyme^®^ TL IM is destroyed when stirred, a fixed bed reactor was selected for the present study which has shown that it can be used up to seven times with only a 10% loss in its activity in this mode of use (Figure 1). Lipozyme^®^ TL IM is also much cheaper than Lipozyme^®^ 435. As DHA accounts for 55.43% of TG at the sn‐2 position, a DHA content as high as 70.3% in the heavy component was unexpected. Therefore, we can infer that Lipozyme^®^ TL IM selectively targeted PA rather than DHA at the sn‐1(3) position due to steric restrictions that block a fatty acid with a longer carbon chain from accessing the active catalyst site for esterification.

Algal oil has a poor solubility in ethanol, which does not favor ethanolysis. However, as the reaction progressed, we observed that the turbidity of the reaction solution gradually disappeared, which greatly facilitated the progress of the reaction. This might have been caused by the accumulation of fatty acids in the MG form acting as surfactants, which helped to mix the oil and ethanol.

The evaporator temperature appeared to be a determining factor influencing the DHA content in the heavy component, because the boiling points of the reaction products were in the order TG > DG > MG > EE and DHA‐EE > PA‐EE. The DHA contents increased with increasing evaporator temperature up to 150°C or above (Table 2). We also found that fatty acids in the EE and MG form could be separated using molecular distillation by increasing the evaporator temperature. When the evaporator temperature reached 150°C, the DHA‐MG was present mostly in the heavy component (79.58%), while DHA‐EE was present mostly in the light component (98.40%). However, when the evaporator temperature reached 180°C, the DHA‐MG was present mostly in the light component (81.68%) (Table 3). Therefore, an evaporator temperature of about 150°C was found to be efficient for separating the EE and MG forms of DHA. Therefore, 150°C was set as the optimum temperature in the present study for excluding the EE form of DHA from the heavy component.

When the evaporator temperature was higher than 150°C, although more saturated fatty acids would be evaporated and collected in the light component, some DHA‐MG would also be evaporated. This explained the inefficiency of further raising the DHA content by increasing the evaporator temperature above 150°C. Our findings showed that enzymatic ethanolysis in tandem with molecular distillation can also be used for the synthesis and separation of DHA‐EE and DHA‐MG by altering the evaporator temperature. In theory, DG and TG can be separated by further increasing the evaporator to a higher temperature but when the temperature was higher than 180°C, we found that the color of the DHA algal oil became dark rather than remaining a light yellow color, making it necessary to use other methods, such as column chromatography, to further separate DG and TG.

In conclusion, the present study has developed an eco‐friendly and simple method combining ethanolysis and molecular distillation to separate saturated oils from algal oil thereby increasing its DHA content from 45.94% to 70.27%.

## CONFLICT OF INTEREST

The authors declare no conflict of interest.

## ETHICAL STATEMENT

This study does not involve any human or animal testing.
